# Bis(*O*,*O*′-diphenethyl dithio­phosphato-κ^2^ *S*,*S*′)bis­(4-methyl­pyridine-κ*N*)nickel(II)

**DOI:** 10.1107/S1600536808020898

**Published:** 2008-07-12

**Authors:** Jian-Shen Feng, Yu Cheng, Li-Ke Zou, Bin Xie, Xiu-Lan Zhang

**Affiliations:** a College of Chemistry and Pharmaceutical Engineering, Sichuan University of Science & Engineering, Zigong, Sichuan 643000, People’s Republic of China

## Abstract

The title complex, [Ni(C_16_H_18_O_2_PS_2_)_2_(C_6_H_7_N)_2_], exhibits a roughly octa­hedral coordination geometry. The Ni^II^ atom lies on an inversion centre and is coordinated by four S atoms of *O*,*O′*-diphenethyl dithio­phosphate mol­ecules and two N atoms of 4-methyl­pyridine mol­ecules. Important geometric data include Ni—N = 2.100 (3) Å, and Ni—S = 2.5101 (10) and 2.4772 (11) Å.

## Related literature

For related literature, see: Allen (2002 ▶); Drew *et al.* (1987 ▶); Harrison *et al.* (1987 ▶); Liu *et al.* (1997 ▶); Li *et al.* (2006 ▶).
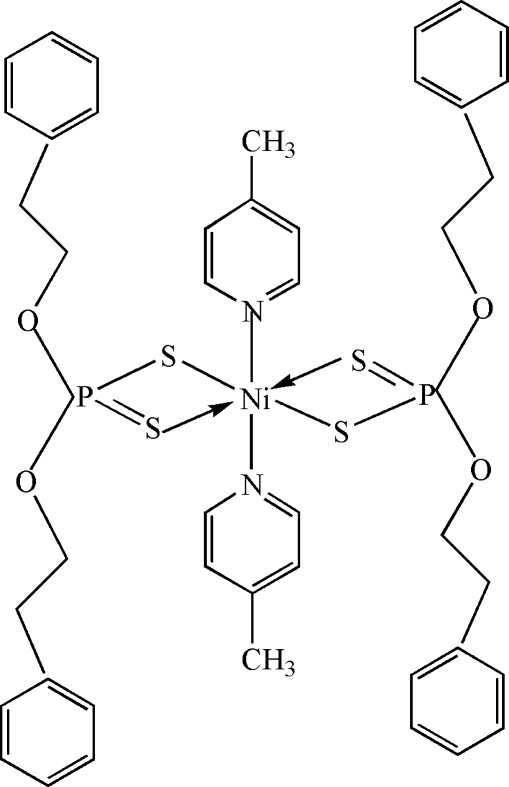


## Experimental

### 

#### Crystal data

[Ni(C_16_H_18_O_2_PS_2_)_2_(C_6_H_7_N)_2_]
*M* *_r_* = 919.77
Monoclinic, 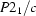

*a* = 12.920 (4) Å
*b* = 17.498 (4) Å
*c* = 10.979 (3) Å
β = 113.05 (3)°
*V* = 2283.9 (12) Å^3^
*Z* = 2
Mo *K*α radiation
μ = 0.72 mm^−1^
*T* = 294 (2) K
0.50 × 0.48 × 0.33 mm

#### Data collection

Enraf–Nonius CAD-4 diffractometer
Absorption correction: spherical (*WinGX*; Farrugia, 1999 ▶) *T*_min_ = 0.715, *T*_max_ = 0.797
4524 measured reflections
4263 independent reflections
2538 reflections with *I* > 2σ(*I*)
*R*_int_ = 0.004
3 standard reflections every 300 reflections intensity decay: 0.3%

#### Refinement

*R*[*F* ^2^ > 2σ(*F* ^2^)] = 0.044
*wR*(*F* ^2^) = 0.125
*S* = 0.98
4263 reflections
263 parameters
H-atom parameters constrained
Δρ_max_ = 0.40 e Å^−3^
Δρ_min_ = −0.41 e Å^−3^

### 

Data collection: *CAD-4 Software* (Enraf–Nonius, 1989 ▶); cell refinement: *CAD-4 Software*; data reduction: *XCAD4* (Harms & Wocadlo, 1995 ▶); program(s) used to solve structure: *SHELXS97* (Sheldrick, 2008 ▶); program(s) used to refine structure: *SHELXL97* (Sheldrick, 2008 ▶); molecular graphics: *ORTEP-3 for Windows* (Farrugia, 1997 ▶); software used to prepare material for publication: *SHELXL97*.

## Supplementary Material

Crystal structure: contains datablocks I, global. DOI: 10.1107/S1600536808020898/dn2365sup1.cif

Structure factors: contains datablocks I. DOI: 10.1107/S1600536808020898/dn2365Isup2.hkl

Additional supplementary materials:  crystallographic information; 3D view; checkCIF report

## Figures and Tables

**Fig. 1.**
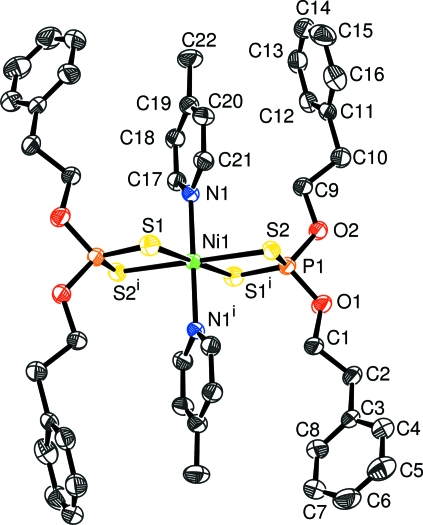
A View of the title complexe showing the atom-labelling scheme. Displacement ellipsoids are drawn at the 30% probability level. H atoms have been omitted for the sake of clarity.[Symmetry code: (i) -x + 1, -y, -z].

## supplementary crystallographic information

### Comment

Interest in the chemistry of metal complexes of *O*,*O'*- dialkyldithiophosphates continues to grow due to extensively employed as anti-oxidants, additives to lubricating oils, flotation reagents, insecticides(Harrison *et al.*,1987; Liu *et al.*, 1997;Li *et al.*,2006). *O*,*O'*-Dialkyldithiophosphates exhibit remarkable variety of forms of coordination to metal (Drew *et al.*,1987). These systems can adopt a variety of molecular and crystal structures, mono-,bi-,tetra-,and polynuclear. We report here the synthesis and crystal structure of Ni[S_2_P(OCH_2_CH_2_Ph)_2_]_2_(NC_5_H_4_CH_3_-4)_2_.

The Ni^II^ atom exhibits a roughly octahedral geometry,and lies on an inversion center (Fig.1). The bond lengths and angles within the complex may be considered normal in comparison with the Cambridge Structural Database results (Allen, 2002).

### Experimental

90 ml hot aqueous solution of Ni(OAc)_2_.4 H_2_O (1.87 g, 7.5 mmol) was added to 90 ml boiling methanol solution of [(PhCH_2_CH_2_O)_2_PS_2_]NH_2_(CH_2_CH_3_)_2_(6.42 g, 15.75 mmol). The mixture was refluxed and stirred for 30 minutes.After cooling to room temperature, the resulting Ni[S_2_P(OCH_2_CH_2_Ph)_2_]_2_ precipitate was collected by filtration and washed with methanol.

0.56 g 4-methylpyridine was added to a solution of Ni[S_2_P(OCH_2_CH_2_Ph)_2_]_2_ (0.72 g, 1 mmol) in 30 ml acetone and 60 ml petroleum ether, then the reaction mixture was concentrated to about 30 ml with a vacuum rotary evaporator. After cooling to room temperature, the pale green precipitate was collected by filtration and washed with petroleum ether.The block crystal was dissovled in a solution of 0.6 g 4-methylpyridine in 30 ml acetone and 20 ml petroleum ether, and the solution was kept at room temperature, green block crystals of Ni[S_2_P(OCH_2_CH_2_Ph)_2_]_2_(NC_5_H_4_CH_3_-4)_2_ were obtained in four weeks.

### Refinement

All H atoms attached to C atom were fixed geometrically and treated as riding with C—H = 0.93 Å (aromatic), 0.97 Å (methylene), 0.96 Å (methyl) and *U*_iso_(H) =1.2*U*_eq_(C, aromatic and methylene) or 1.5*U*_eq_ (C, methyl).

### Crystal data

**Table:**

[Ni(C_16_H_18_O_2_PS_2_)_2_(C_6_H_7_N)_2_]	*F*_000_ = 964
*M**_r_* = 919.77	*D*_x_ = 1.337 Mg m^−^^3^
Monoclinic, *P*2_1_/*c*	Mo *K*α radiation λ = 0.71073 Å
Hall symbol: -P 2ybc	Cell parameters from 25 reflections
*a* = 12.920 (4) Å	θ = 4.7–7.8º
*b* = 17.498 (4) Å	µ = 0.72 mm^−^^1^
*c* = 10.979 (3) Å	*T* = 294 (2) K
β = 113.05 (3)º	Block, green
*V* = 2283.9 (12) Å^3^	0.50 × 0.48 × 0.33 mm
*Z* = 2	

### Data collection

**Table:**

Enraf–Nonius CAD-4 diffractometer	*R*_int_ = 0.004
Radiation source: fine-focus sealed tube	θ_max_ = 25.6º
Monochromator: graphite	θ_min_ = 1.7º
*T* = 294(2) K	*h* = −15→6
ω/2θ scans	*k* = −21→0
Absorption correction: for a sphere(WINGX; Farrugia, 1999)	*l* = −12→13
*T*_min_ = 0.715, *T*_max_ = 0.797	3 standard reflections
4524 measured reflections	every 300 reflections
4263 independent reflections	intensity decay: 0.3%
2538 reflections with *I* > 2σ(*I*)	

### Refinement

**Table:**

Refinement on *F*^2^	Secondary atom site location: difference Fourier map
Least-squares matrix: full	Hydrogen site location: inferred from neighbouring sites
*R*[*F*^2^ > 2σ(*F*^2^)] = 0.044	H-atom parameters constrained
*wR*(*F*^2^) = 0.125	*w* = 1/[σ^2^(*F*_o_^2^) + (0.072*P*)^2^] where *P* = (*F*_o_^2^ + 2*F*_c_^2^)/3
*S* = 0.99	(Δ/σ)_max_ < 0.001
4263 reflections	Δρ_max_ = 0.40 e Å^−^^3^
263 parameters	Δρ_min_ = −0.41 e Å^−^^3^
Primary atom site location: structure-invariant direct methods	Extinction correction: none

### Special details

**Table:**

Geometry. All e.s.d.'s (except the e.s.d. in the dihedral angle between two l.s. planes) are estimated using the full covariance matrix. The cell e.s.d.'s are taken into account individually in the estimation of e.s.d.'s in distances, angles and torsion angles; correlations between e.s.d.'s in cell parameters are only used when they are defined by crystal symmetry. An approximate (isotropic) treatment of cell e.s.d.'s is used for estimating e.s.d.'s involving l.s. planes.
Refinement. Refinement of *F*^2^ against ALL reflections. The weighted *R*-factor *wR* and goodness of fit *S* are based on *F*^2^, conventional *R*-factors *R* are based on *F*, with *F* set to zero for negative *F*^2^. The threshold expression of *F*^2^ > σ(*F*^2^) is used only for calculating *R*-factors(gt) *etc*. and is not relevant to the choice of reflections for refinement. *R*-factors based on *F*^2^ are statistically about twice as large as those based on *F*, and *R*- factors based on ALL data will be even larger.

### Fractional atomic coordinates and isotropic or equivalent isotropic displacement parameters (Å^2^)

**Table:**

	*x*	*y*	*z*	*U*_iso_*/*U*_eq_	
Ni1	0.5000	0.0000	0.0000	0.04415 (18)	
S1	0.37867 (8)	0.09900 (5)	0.04468 (9)	0.0600 (3)	
S2	0.68569 (7)	0.06097 (5)	0.11775 (9)	0.0560 (3)	
P1	0.74567 (7)	−0.03034 (6)	0.06089 (9)	0.0566 (3)	
O1	0.8408 (2)	−0.07200 (17)	0.1803 (2)	0.0721 (8)	
O2	0.82597 (19)	−0.00827 (16)	−0.0117 (2)	0.0738 (8)	
N1	0.4851 (2)	0.05930 (16)	−0.1723 (3)	0.0471 (6)	
C1	0.8201 (3)	−0.0924 (2)	0.2958 (3)	0.0643 (10)	
H1A	0.7570	−0.1274	0.2720	0.077*	
H1B	0.8024	−0.0471	0.3350	0.077*	
C2	0.9244 (3)	−0.1296 (3)	0.3921 (4)	0.0693 (11)	
H2A	0.9359	−0.1780	0.3564	0.083*	
H2B	0.9887	−0.0972	0.4049	0.083*	
C3	0.9159 (3)	−0.1428 (2)	0.5224 (4)	0.0599 (9)	
C4	0.9828 (4)	−0.1032 (3)	0.6319 (4)	0.0779 (12)	
H4	1.0354	−0.0690	0.6252	0.094*	
C5	0.9745 (5)	−0.1126 (3)	0.7503 (5)	0.1013 (16)	
H5	1.0213	−0.0851	0.8237	0.122*	
C6	0.8984 (5)	−0.1618 (4)	0.7619 (5)	0.1083 (19)	
H6	0.8916	−0.1674	0.8427	0.130*	
C7	0.8314 (4)	−0.2035 (3)	0.6549 (6)	0.111 (2)	
H7	0.7791	−0.2376	0.6627	0.133*	
C8	0.8416 (4)	−0.1947 (3)	0.5345 (4)	0.0880 (14)	
H8	0.7978	−0.2241	0.4621	0.106*	
C9	0.7811 (3)	0.0340 (3)	−0.1333 (4)	0.0786 (13)	
H9A	0.7260	0.0707	−0.1300	0.094*	
H9B	0.7443	−0.0005	−0.2068	0.094*	
C10	0.8743 (3)	0.0741 (3)	−0.1521 (4)	0.0792 (12)	
H10A	0.9305	0.0371	−0.1509	0.095*	
H10B	0.9094	0.1092	−0.0790	0.095*	
C11	0.8351 (3)	0.1179 (2)	−0.2803 (3)	0.0565 (9)	
C12	0.7967 (3)	0.0800 (2)	−0.3984 (4)	0.0656 (10)	
H12	0.7931	0.0269	−0.3995	0.079*	
C13	0.7637 (3)	0.1192 (3)	−0.5139 (4)	0.0856 (14)	
H13	0.7388	0.0922	−0.5931	0.103*	
C14	0.7658 (4)	0.1953 (4)	−0.5172 (6)	0.1012 (19)	
H14	0.7428	0.2208	−0.5978	0.121*	
C15	0.8018 (4)	0.2354 (3)	−0.4018 (7)	0.1067 (19)	
H15	0.8026	0.2886	−0.4026	0.128*	
C16	0.8375 (4)	0.1958 (3)	−0.2827 (5)	0.0807 (13)	
H16	0.8633	0.2228	−0.2034	0.097*	
C17	0.4546 (3)	0.0236 (2)	−0.2882 (3)	0.0552 (9)	
H17	0.4386	−0.0284	−0.2919	0.066*	
C18	0.4457 (3)	0.0600 (2)	−0.4025 (4)	0.0642 (10)	
H18	0.4229	0.0329	−0.4815	0.077*	
C19	0.4706 (3)	0.1367 (3)	−0.4005 (4)	0.0674 (11)	
C20	0.5011 (3)	0.1735 (2)	−0.2822 (4)	0.0691 (11)	
H20	0.5177	0.2254	−0.2764	0.083*	
C21	0.5072 (3)	0.1338 (2)	−0.1714 (4)	0.0588 (9)	
H21	0.5278	0.1602	−0.0919	0.071*	
C22	0.4675 (4)	0.1771 (3)	−0.5222 (5)	0.1025 (17)	
H22A	0.4301	0.2254	−0.5301	0.154*	
H22B	0.4275	0.1464	−0.5987	0.154*	
H22C	0.5430	0.1854	−0.5156	0.154*	

### Atomic displacement parameters (Å^2^)

**Table:**

	*U*^11^	*U*^22^	*U*^33^	*U*^12^	*U*^13^	*U*^23^
Ni1	0.0442 (3)	0.0490 (3)	0.0422 (3)	−0.0013 (3)	0.0201 (3)	0.0022 (3)
S1	0.0643 (6)	0.0611 (6)	0.0612 (6)	0.0078 (5)	0.0317 (5)	0.0007 (5)
S2	0.0515 (5)	0.0646 (6)	0.0510 (5)	−0.0106 (4)	0.0190 (4)	0.0018 (4)
P1	0.0450 (5)	0.0802 (7)	0.0490 (5)	0.0067 (5)	0.0233 (4)	0.0164 (5)
O1	0.0545 (14)	0.111 (2)	0.0570 (15)	0.0208 (14)	0.0285 (12)	0.0289 (14)
O2	0.0479 (13)	0.123 (2)	0.0581 (15)	0.0103 (14)	0.0287 (12)	0.0319 (15)
N1	0.0449 (15)	0.0522 (17)	0.0490 (16)	0.0003 (13)	0.0234 (13)	0.0049 (13)
C1	0.053 (2)	0.089 (3)	0.056 (2)	0.0092 (19)	0.0264 (18)	0.023 (2)
C2	0.051 (2)	0.094 (3)	0.059 (2)	0.013 (2)	0.0184 (19)	0.021 (2)
C3	0.049 (2)	0.069 (2)	0.055 (2)	0.0095 (19)	0.0131 (17)	0.0187 (19)
C4	0.073 (3)	0.089 (3)	0.065 (3)	−0.002 (2)	0.021 (2)	0.011 (2)
C5	0.119 (4)	0.110 (4)	0.062 (3)	0.007 (3)	0.021 (3)	0.005 (3)
C6	0.106 (4)	0.157 (5)	0.067 (3)	0.021 (4)	0.040 (3)	0.040 (4)
C7	0.075 (3)	0.154 (5)	0.097 (4)	−0.013 (3)	0.028 (3)	0.058 (4)
C8	0.078 (3)	0.112 (4)	0.062 (3)	−0.020 (3)	0.015 (2)	0.023 (3)
C9	0.056 (2)	0.127 (4)	0.057 (2)	0.003 (2)	0.0274 (19)	0.035 (2)
C10	0.063 (2)	0.120 (4)	0.056 (2)	−0.005 (2)	0.025 (2)	0.020 (2)
C11	0.0480 (19)	0.079 (3)	0.051 (2)	−0.0007 (18)	0.0296 (17)	0.0078 (19)
C12	0.057 (2)	0.080 (3)	0.061 (2)	−0.0134 (19)	0.0246 (19)	−0.007 (2)
C13	0.064 (3)	0.139 (5)	0.053 (3)	−0.014 (3)	0.023 (2)	0.004 (3)
C14	0.064 (3)	0.156 (6)	0.086 (4)	0.005 (3)	0.033 (3)	0.056 (4)
C15	0.101 (4)	0.074 (3)	0.170 (6)	0.022 (3)	0.080 (4)	0.042 (4)
C16	0.085 (3)	0.084 (3)	0.088 (3)	−0.010 (3)	0.050 (3)	−0.023 (3)
C17	0.054 (2)	0.063 (2)	0.051 (2)	0.0008 (17)	0.0234 (17)	−0.0012 (18)
C18	0.058 (2)	0.092 (3)	0.048 (2)	0.007 (2)	0.0268 (18)	0.007 (2)
C19	0.055 (2)	0.086 (3)	0.069 (3)	0.012 (2)	0.033 (2)	0.025 (2)
C20	0.071 (3)	0.063 (2)	0.078 (3)	0.002 (2)	0.033 (2)	0.020 (2)
C21	0.061 (2)	0.058 (2)	0.061 (2)	−0.0007 (18)	0.0280 (19)	0.0034 (19)
C22	0.098 (3)	0.134 (4)	0.090 (3)	0.019 (3)	0.052 (3)	0.056 (3)

### Geometric parameters (Å, °)

**Table:**

Ni1—N1^i^	2.100 (3)	C8—H8	0.9300
Ni1—N1	2.100 (3)	C9—C10	1.476 (5)
Ni1—S2	2.4772 (11)	C9—H9A	0.9700
Ni1—S2^i^	2.4772 (11)	C9—H9B	0.9700
Ni1—S1	2.5101 (10)	C10—C11	1.507 (5)
Ni1—S1^i^	2.5101 (10)	C10—H10A	0.9700
S1—P1^i^	1.9772 (15)	C10—H10B	0.9700
S2—P1	1.9803 (15)	C11—C16	1.363 (6)
P1—O1	1.581 (3)	C11—C12	1.365 (5)
P1—O2	1.584 (2)	C12—C13	1.355 (6)
P1—S1^i^	1.9772 (15)	C12—H12	0.9300
O1—C1	1.440 (4)	C13—C14	1.332 (7)
O2—C9	1.435 (4)	C13—H13	0.9300
N1—C17	1.332 (4)	C14—C15	1.362 (7)
N1—C21	1.333 (4)	C14—H14	0.9300
C1—C2	1.497 (5)	C15—C16	1.390 (7)
C1—H1A	0.9700	C15—H15	0.9300
C1—H1B	0.9700	C16—H16	0.9300
C2—C3	1.495 (5)	C17—C18	1.371 (5)
C2—H2A	0.9700	C17—H17	0.9300
C2—H2B	0.9700	C18—C19	1.378 (5)
C3—C4	1.363 (5)	C18—H18	0.9300
C3—C8	1.365 (5)	C19—C20	1.362 (5)
C4—C5	1.356 (6)	C19—C22	1.498 (5)
C4—H4	0.9300	C20—C21	1.376 (5)
C5—C6	1.350 (7)	C20—H20	0.9300
C5—H5	0.9300	C21—H21	0.9300
C6—C7	1.365 (7)	C22—H22A	0.9600
C6—H6	0.9300	C22—H22B	0.9600
C7—C8	1.387 (6)	C22—H22C	0.9600
C7—H7	0.9300		
			
N1^i^—Ni1—N1	180.0	C3—C8—C7	120.0 (5)
N1^i^—Ni1—S2	90.83 (8)	C3—C8—H8	120.0
N1—Ni1—S2	89.17 (8)	C7—C8—H8	120.0
N1^i^—Ni1—S2^i^	89.17 (8)	O2—C9—C10	108.7 (3)
N1—Ni1—S2^i^	90.83 (8)	O2—C9—H9A	110.0
S2—Ni1—S2^i^	180.0	C10—C9—H9A	110.0
N1^i^—Ni1—S1	90.50 (8)	O2—C9—H9B	110.0
N1—Ni1—S1	89.50 (8)	C10—C9—H9B	110.0
S2—Ni1—S1	98.75 (4)	H9A—C9—H9B	108.3
S2^i^—Ni1—S1	81.25 (4)	C9—C10—C11	112.3 (3)
N1^i^—Ni1—S1^i^	89.50 (8)	C9—C10—H10A	109.2
N1—Ni1—S1^i^	90.50 (8)	C11—C10—H10A	109.2
S2—Ni1—S1^i^	81.25 (4)	C9—C10—H10B	109.2
S2^i^—Ni1—S1^i^	98.75 (4)	C11—C10—H10B	109.2
S1—Ni1—S1^i^	180.0	H10A—C10—H10B	107.9
P1^i^—S1—Ni1	83.82 (5)	C16—C11—C12	118.1 (4)
P1—S2—Ni1	84.64 (4)	C16—C11—C10	121.7 (4)
O1—P1—O2	94.61 (13)	C12—C11—C10	120.3 (4)
O1—P1—S1^i^	113.28 (13)	C13—C12—C11	120.4 (4)
O2—P1—S1^i^	113.27 (12)	C13—C12—H12	119.8
O1—P1—S2	112.56 (12)	C11—C12—H12	119.8
O2—P1—S2	112.10 (12)	C14—C13—C12	121.9 (5)
S1^i^—P1—S2	110.28 (6)	C14—C13—H13	119.0
C1—O1—P1	119.5 (2)	C12—C13—H13	119.0
C9—O2—P1	119.2 (2)	C13—C14—C15	119.5 (5)
C17—N1—C21	116.6 (3)	C13—C14—H14	120.2
C17—N1—Ni1	121.2 (2)	C15—C14—H14	120.2
C21—N1—Ni1	122.2 (2)	C14—C15—C16	119.0 (5)
O1—C1—C2	107.7 (3)	C14—C15—H15	120.5
O1—C1—H1A	110.2	C16—C15—H15	120.5
C2—C1—H1A	110.2	C11—C16—C15	121.0 (4)
O1—C1—H1B	110.2	C11—C16—H16	119.5
C2—C1—H1B	110.2	C15—C16—H16	119.5
H1A—C1—H1B	108.5	N1—C17—C18	122.9 (4)
C3—C2—C1	111.1 (3)	N1—C17—H17	118.5
C3—C2—H2A	109.4	C18—C17—H17	118.5
C1—C2—H2A	109.4	C17—C18—C19	120.2 (4)
C3—C2—H2B	109.4	C17—C18—H18	119.9
C1—C2—H2B	109.4	C19—C18—H18	119.9
H2A—C2—H2B	108.0	C20—C19—C18	117.0 (4)
C4—C3—C8	118.7 (4)	C20—C19—C22	121.6 (4)
C4—C3—C2	120.4 (4)	C18—C19—C22	121.4 (4)
C8—C3—C2	120.9 (4)	C19—C20—C21	120.0 (4)
C5—C4—C3	121.5 (5)	C19—C20—H20	120.0
C5—C4—H4	119.2	C21—C20—H20	120.0
C3—C4—H4	119.2	N1—C21—C20	123.3 (4)
C6—C5—C4	120.1 (5)	N1—C21—H21	118.4
C6—C5—H5	120.0	C20—C21—H21	118.4
C4—C5—H5	120.0	C19—C22—H22A	109.5
C5—C6—C7	120.0 (5)	C19—C22—H22B	109.5
C5—C6—H6	120.0	H22A—C22—H22B	109.5
C7—C6—H6	120.0	C19—C22—H22C	109.5
C6—C7—C8	119.7 (5)	H22A—C22—H22C	109.5
C6—C7—H7	120.1	H22B—C22—H22C	109.5
C8—C7—H7	120.1		

Symmetry codes: (i) −*x*+1, −*y*, −*z*.
